# *eIF4E*基因在非小细胞肺癌中的表达及意义

**DOI:** 10.3779/j.issn.1009-3419.2010.12.10

**Published:** 2010-12-20

**Authors:** 波 张, 成楚 朱, 保富 陈, 霞 章, 敏华 叶, 爱芬 林

**Affiliations:** 317000 台州，浙江省台州医院胸外科 Department of Thoracic Surgery, Taizhou Hospital Affiliated to Wenzhou Medical College, Taizhou 317000, China

**Keywords:** 肺肿瘤, 免疫组织化学, 真核细胞翻译起始因子, Lung neoplasms, Immunohistochemistry, Eukaryotic initiation factor-4E

## Abstract

**背景与目的:**

以往研究表明，真核细胞翻译起始因子（eukaryotic initiation factor-4E, eIF4E）在多种实体肿瘤中高表达，与恶性肿瘤发生、浸润和转移密切相关。本研究旨在采用免疫组化方法研究eIF4E在非小细胞肺癌（non-small cell lung cancer, NSCLC）中的表达及意义。

**方法:**

70例NSCLC石蜡标本制成组织芯片，以19例癌旁组织、20例正常肺组织为对照，均采用Envision两步法进行免疫组化检测。

**结果:**

eIF4E在NSCLC中的阳性表达率高于在癌旁组织和正常肺组织的表达率；eIF4E在淋巴结转移组阳性表达率高于在无淋巴结转移组中表达率；eIF4E在低分化组中阳性表达率高于在中高分化组中表达率。eIF4E阳性表达与患者性别、年龄、肿块大小、吸烟情况无统计学差异（*P* > 0.05）。

**结论:**

eIF4E在NSCLC中有较高的表达率，且与淋巴结转移密切相关，提示NSCLC的发生、侵袭和转移可能与eIF4E相关，eIF4E有可能作为肺癌新的标志物以及评估肺癌进展客观指标。

## Abstract (en)

**Background and objective:**

Precious studies proven that the overexpression of eukaryotic translation initiation factor 4E (eIF4E) in a variety of solid tumors has linked with malignant tumorigenesis, invasion and metastasis. The aim of this work is to explore the expression of eIF4E and its clinical significance in non-small cell lung cancer (NSCLC) through immunohistochemical method.

**Methods:**

Seventy NSCLC specimens were constructed into tissue microarray, simultaneously took 19 cases adjacent tissues and 20 cases normal lung tissue as control, all performed using immunohistochemistry (Envision).

**Results:**

eIF4E expression in NSCLC was significantly higher than that in adjacent tissues and normal lung tissues, statistically significant difference between the groups. The eIF4E positive expression in lymph node metastasis group was significantly higher than that in no-lymph node metastasis. eIF4E protein positive expression of WD/MD cancer tissue was significantly lower than those in PD/ND cancer tissue. There were no significant differences of positive expression of eIF4E with the tumor diameter, age, smoking status, gender of NSCLC patients (*P* > 0.05).

**Conclusion:**

There were highly over expression rate of eIF4E, and closely correlated with lymphnode metastasis in NSCLC, which implies that the eIF4E expression may be related to tumorgenesis, invasion and metastasis of lung cancer. eIF4E may be a new marker for lung cancer and objective indicators assessing the development of lung cancer.

目前在世界范围内，肺癌已成为癌症死亡的主要原因，严重危害人类健康和生命^[1]^。这与肿瘤细胞侵袭性和转移性的生物学特征密切相关，然而对其侵袭和转移机制的研究仍是一大难题。因此，有必要从多角度、多方位去研究。真核细胞翻译起始因子（eukaryotic Initiation factor-4E, eIF4E），是真核细胞翻译起始和调控的核心成分，在蛋白质翻译起始过程中发挥重要作用。研究表明在多种实体肿瘤高表达，与肿瘤的侵袭和转移密切相关。分析国内外文献，其在非小细胞肺癌（non-small cell lung cancer, NSCLC）中研究报道甚少。本研究采用组织芯片技术结合免疫组化研究eIF4E在非小细胞肺癌组织、癌旁组织和正常肺组织中表达差异，分析其在NSCLC发生、侵袭和转移中的作用。

## 1 材料与方法

### 1.1 研究对象

组织标本均来自温州医学院附属台州医院人体组织标本库存档蜡块，整理2004年3月-2007年3月行肺癌根治术的NSCLC石蜡包埋标本70例，所有标本的患者术前均未接受化疗及放疗。所有病例行肺癌根治术（系统淋巴结清扫：包括隆突下淋巴结在内的纵膈淋巴结≥3组，肺内及肺门淋巴结≥3组）。其中男性52例，女性18例；年龄35岁-80岁，平均58.3岁；淋巴结转移37例，无淋巴结转移33例。标本按WHO肺癌病理组织学分型：鳞癌36例，腺癌34例。组织分化程度：低分化（包括未分化）27例，中分化25例，高分化18例。肿瘤TNM分期：T1期18例，T2期41例，T3期9例，T4期2例。N分期：N0期33例，N1期28例，N2期9例。

另取NSCLC癌旁组织（距肿块边缘1.5 cm的肺组织）19例，正常组织（距肿块边缘5 cm正常肺组织）20例。所有病例均有完整的临床和病理资料，术前均未接受放疗及化疗。

### 1.2 实验试剂与方法

鼠抗人eIF4E抗体购于Acris Antibodies GmbH公司，Envision免疫组化试剂盒、DAB显色试剂盒均购于北京中山生物技术有限公司。采用免疫组化Envision两步法技术，DAB显色，以TBS代替一抗作阴性对照，以乳腺癌标本作阳性对照。

### 1.3 结果分析和判定

所有染色标本均由两位病理医生互盲下进行免疫组化评价。判定标准^[2]^：切片中阳性细胞率及染色反应强度作为分级标准。以细胞浆染色强度分别计分：无着色为0分，淡黄染色为1分，棕黄色为2分，棕褐色/胞浆内棕黑色颗粒为3分；以细胞阳性范围分别计分： < 5%为0分，6%-25%为1分，26%-50%为2分，51%-75%为3分， > 75%为4分；再将染色强度与阳性细胞率作乘积分级： > 3分为（+），6分-9分为（++），9分-12分为（+++），凡（+）或（+）以上者均为阳性表达。

### 1.4 统计学分析

采用SPSS 13.0统计软件包处理数据，组间资料比较采用*χ*^2^检验，以*P* < 0.05为差异有统计学意义。

## 2 结果

### 2.1 eIF4E染色结果

本组中全部阴性对照均无阳性染色。eIF4E主要定位于肺癌组织的癌细胞膜和/或细胞浆中，呈棕黄色颗粒样物（图 1）。

**1:**
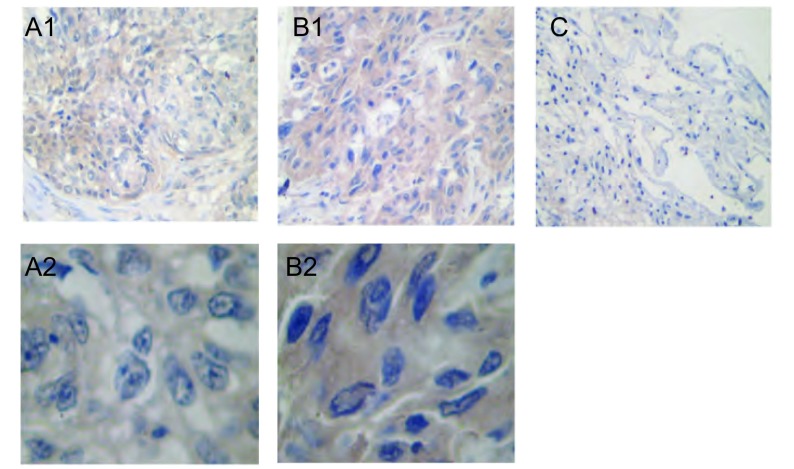
eIF4E在不同肺组织中的表达（A1，B1，C×100；A2，B2×400）。A：eIF4E在腺癌组织中阳性表达；B：eIF4E在鳞癌组织中阳性表达；C：eIF4E在正常组织中无阳性表达。 Expression of eIF4E in squamous cell carcinoma, adenocarcinoma and normal tissue (A1, B1, C×100; A2, B2×400). A: positive expression of eIF4E in adenocarcinoma tissue; B: positive expression of eIF4E in squamous cell carcinoma tissue; C: No positive expression of eIF4E in normal tissue.

### 2.2 eIF4E在NSCLC中的表达

eIF4E在NSCLC中的阳性表达率为81.4%（57/70），eIF4E在癌旁组织以及正常肺组织中的阳性表达率分别为31.6%（6/19）、15.0%（3/20）（表 1），组间差异具有统计学意义（*P* < 0.05）。

**1:** eIF4E在不同肺组织中的表达情况 The expression of eIF4E protein in different lung tissues

Group	Entire sample	elF4E expression				Positive ratio (%)	*χ*^2^	*P*
		(-)	(+)	(++)	(+++)			
Lung cancer tissue	70	13	5	9	43	81.4		
Para-cancerous tissue	19	13	4	1	1	31.6	36.822	< 0.001
Normal pulmonary tissue	20	17	2	1	0	15.0		

### 2.3 eIF4E的表达与NSCLC临床病理特征的关系

淋巴结转移组eIF4E的阳性表达率为91.8%（34/37）；无淋巴结转移组eIF4E的阳性表达率为69.7%（23/33）；组间差异具有统计学意义（*P* < 0.05）。高中分化组和差分化组组间eIF4E的阳性表达率分别为72.1%（31/43）、96.2%（26/27），组间差异具有统计学意义（*P* < 0.05）；eIF4E在腺癌和鳞癌中阳性表达率分别为91.2%（31/34）、72.2%（26/36），组间差异具有统计学意义（*P* < 0.05）。在肿块大小上，直径≤3 cm者和 > 3 cm者，eIF4E的阳性表达率分别为69.2%（14/19）、84.2%（43/51），组间差异无统计学意义（*P* > 0.05）；eIF4E在男性/女性患者的阳性表达率分别为84.6%（44/52）和72.2%（13/18），差异无统计学意义（*P* > 0.05）；在 > 55岁或≤55岁的患者中，eIF4E的阳性表达率分别为80.4%（37/46）和83.3%（20/24），差异无统计学意义（*P* > 0.05）；eIF4E在吸烟者和不吸烟者中阳性表达率分别为79.2%（38/48）、86.4%（19/22），差异无统计学意义（*P* > 0.05）（表 2）。

**2:** eIF4E的表达水平与非小细胞肺癌临床病理特征的关系 Relationship between eIF4E protein expression and clinicopathological characteristics non-small lung cancer

Varial/Category	Entire Sample (*n*=70)	elF4E expression		Positive ratio (%)	*χ*^2^	*P* value
		Negative	Positive			
Age (years)						
≥55	46	9	37	80.4		
< 55	24	4	20	83.3	0.088	0.767
Gender						
Male	52	8	44	84.6		
Female	18	5	13	72.2	0.662	0.416
Smoking history						
Ever	48	10	38	79.2		
Never	22	3	19	86.4	0.150	0.698
Tumor size (cm)						
< 3	19	5	14	69.2		
≥3	51	8	43	84.2	0.451	0.502
Histopathology						
SCC	36	10	26	72.2		
ADC	34	3	31	91.2	4.154	0.042
Differentiation						
WD/MD	43	12	31	72.1		
PD/ND	27	1	26	96.2	6.425	0.011
Lymph node						
No-metastasis group	33	10	23	69.7		
Metastasis group	37	3	34	91.8	5.682	0.017
SCC: squamous cell carcinoma; ADC: adenocarcinoma; WD: well differentiation; MD: moderately differentiation; PD: poorly differentiation; ND: undifferentiation.						

## 3 讨论

肺癌是严重危害人类生命和健康的常见疾病，发病率和死亡率呈逐年上升趋势，而NSCLC占肺癌的80%。近年来，随着细胞生物学和分子生物学的进展，对肺癌发病、侵袭和转移机制的研究取得长足进步，但肺癌患者生存率未得到明显提高。肺癌是多种癌基因和抑癌基因改变和多阶段变化所致的独特生物学行为，有必要从分子生物学角度寻找肺癌诊断和预后的分子指标，研究探讨肺癌分子发病、侵袭和转移机制。eIF4E在蛋白质翻译起始过程中具独特生物学作用，在多种实体肿瘤中高表达，目前受到国内外学者关注。

eIF4E是真核细胞翻译起始和调控的核心成分，在蛋白翻译的起始过程中发挥着重要作用。蛋白翻译的调节紊乱是恶性肿瘤转化和发展过程中非常重要的环节，研究^[3]^发现eIF4E在乳腺癌、结肠癌、脑胶质瘤、黑色素瘤、前列腺癌等肿瘤呈高表达，增加肿瘤血管生成以及播散机率，与肿瘤的进展和预后密切相关。Nathan等^[4]^研究发现，随Akt/mTOR通路信号因子的增加，头颈部癌中eIF4E表达也增加，且雷帕霉素可以潜在的用于治疗eIF4E手术切缘阳性的患者。Robert等^[5]^研究认为反义RNA eIF4E可作为头颈肿瘤患者特别是手术切缘eIF4E阳性患者的辅助治疗手段。在小鼠正常组织和人体肿瘤移植物中，用第二代反义寡核苷酸（ASO）能有效下调肿瘤中eIF4E的含量及蛋白质的表达，可减少受eIF4E调节的蛋白（VEGF、Cyclin D1、cmyc、Bcl-2和Survivin等）的表达，诱导细胞凋亡，阻断内皮细胞的形成，抑制肿瘤生长和血管生成^[6]^。因此，eIF4E有望成为人类实体瘤新的标志物和治疗靶点，但目前对eIF4E与肺癌的研究报道甚少。

多数文献^[7-15]^报道eIF4E在NSCLC中有较高表达率，且与癌肿进展相关，认为eIF4E表达越高者，预后越差，为同淋巴转移和血性转移一样，eIF4E是肺腺癌患者预后的独立因素。本研究经免疫组化半定量方法分析发现，eIF4E在肺癌组织中的阳性表达率较高（81.4%, 57/70），明显高于癌旁组织和正常肺组织，提示eIF4E可能参与肺癌发生、发展过程，表明eIF4E有可能作为NSCLC新的肿瘤标志物。本实验发现，eIF4E表达与年龄、性别、肿瘤大小无关，但eIF4E在肺腺癌中的表达高于在肺鳞癌中表达；肺癌肿瘤分化程度越低，eIF4E的表达越高；这与文献^[7, 8]^报道相一致，意味着eIF4E表达高者，肺肿瘤恶性度相对越高，提示预后差^[9, 10]^。肿瘤侵袭和转移是研究者较为关心的，我们将癌标本根据有无淋巴结转移分为淋巴结转移组和无淋巴结转移组，发现淋巴结转移者中eIF4E的阳性表达明显要高于无淋巴结转移者，差异具有统计学意义。这表明eIF4E在NSCLC的淋巴结转移过程中扮演重要角色，可作为临床预测肺癌早期淋巴结转移指标，为临床诊治提供有价值指导意义。

研究证实eIF4E在多种实体肿瘤高表达，与肿瘤的侵袭和转移密切相关。本研究证实eIF4E在NSCLC中高表达，参与NSCLC的发生和淋巴结转移，可能作为肺癌新的标志物以及评估肺癌进展客观指标。但eIF4E在NSCLC发生、侵袭和转移过程中的作用机理有待进一步研究。
